# SARS-CoV-2 incidence in secondary schools; the role of national and school-initiated COVID-19 measures

**DOI:** 10.1186/s12889-023-16146-0

**Published:** 2023-06-27

**Authors:** L. Jonker, K. J. Linde, A. R. de Boer, E. Ding, D. Zhang, M. L. A. de Hoog, S. Herfst, D. J. J. Heederik, P. L. A. Fraaij, P. M. Bluyssen, I. M. Wouters, P. C. J. L. Bruijning-Verhagen

**Affiliations:** 1 Julius Center for Health Sciences and Primary Care, UMC Utrecht, Universiteitsweg 100, 3584 CG Utrecht, The Netherlands; 2 Institute for Risk Assessment Sciences, Utrecht University, Yalelaan 2, 3584 CM Utrecht, the Netherlands; 3 Faculty of Architecture and the Built Environment, Delft University of Technology, Julianalaan 134, 2628 BL Delft, the Netherlands; 4 Department of Viroscience, Erasmus MC, Dr. Molewaterplein 50, 3015 GE, 3000 CA Rotterdam, Netherlands; 5 Department of Pediatrics, Erasmus MC, Dr. Molewaterplein 50, 3015 GE, 3000 CA Rotterdam, Netherlands

**Keywords:** SARS-CoV-2, COVID-19 measures, Secondary schools, Air contamination, CO_2_ concentration

## Abstract

**Introduction:**

Our aim was to gain insight into the effect of COVID-19 measures on SARS-CoV-2 incidence in secondary schools and the association with classroom CO_2_ concentration and airborne contamination.

**Methods:**

Between October 2020—June 2021, 18 schools weekly reported SARS-CoV-2 incidence and completed surveys on school-initiated COVID-19 measures (e.g. improving hygiene or minimizing contacts). CO_2_ was measured in occupied classrooms twice, and SARS-CoV-2 air contamination longitudinally using electrostatic dust collectors (EDC) and analyzed using RT-qPCR. National COVID-19 policy measures varied during pre-lockdown, lockdown and post-lockdown periods. During the entire study, schools were recommended to improve ventilation. SARS-CoV-2 incidence rate ratios (IRR) were estimated by Generalized Estimating Equation (GEE) models.

**Results:**

During 18 weeks follow-up (range: 10–22) SARS-CoV-2 school-incidence decreased during national lockdown (adjusted IRR: 0.41, 95%CI: 0.21–0.80) and post-lockdown (IRR: 0.60, 0.39–0.93) compared to pre-lockdown. School-initiated COVID-19 measures had no additional effect. Pre-lockdown, IRRs per 10% increase in time CO_2_ exceeded 400, 550 and 800 ppm above outdoor level respectively, were 1.08 (1.00–1.16), 1.10 (1.02–1.19), and 1.08 (0.95–1.22). Post-lockdown, CO_2-_concentrations were considerably lower and not associated with SARS-CoV-2 incidence. No SARS-CoV-2 RNA was detected in any of the EDC samples.

**Conclusion:**

During a period with low SARS-CoV-2 population immunity and increased attention to ventilation, with CO_2_ levels most of the time below acceptable thresholds, only the national policy during and post-lockdown of reduced class-occupancy, stringent quarantine, and contact testing reduced SARS-CoV-2 incidence in Dutch secondary schools. Widespread SARS-CoV-2 air contamination could not be demonstrated in schools under the prevailing conditions during the study.

**Supplementary Information:**

The online version contains supplementary material available at 10.1186/s12889-023-16146-0.

## Introduction

The conditions in secondary schools are generally considered favorable for the transmission of SARS-CoV-2. Large numbers of students are accommodated in often closely spaced classrooms, while ventilation may be suboptimal and high contact rates among students are common. Furthermore, adolescents often experience asymptomatic or mildly symptomatic SARS-CoV-2 infection and may therefore continue their daily activities longer while being unaware they are infectious [1]. During the COVID-19 pandemic, governments were forced to take far-reaching mitigation measures to control spread such as stringent class quarantine policies, frequent testing, wearing masks, partial in-person learning, or even complete school closure. These measures were highly disruptive for education and student well-being and alternative, less disruptive but effective approaches are urgently needed [2, 3]. Improved ventilation has been suggested as an alternative to reduce transmission mainly via the aerosol-route and improved hygiene may help control transmission via (in)direct contact. Measures in schools aiming at reducing crowding in heavily visited areas such as canteens and corridors or at minimizing student–student and student-staff mixing through cohorting have also been advised [4]. However, the evidence base for most of these interventions is weak and two years into the pandemic, uncertainty remains about their effectiveness [4].

To gain further insight, we performed a prospective study in Dutch secondary schools between October 2020 and June 2021. We examined the association between SARS-CoV-2 incidence within schools and the effect of national COVID-19 policy and school-initiated COVID-19 measures on the transmission of SARS-CoV-2 in secondary schools, as well as the role of CO_2_ concentration and airborne contamination in this setting.

## Methods

### Study design and study population

**Fig. 1:**
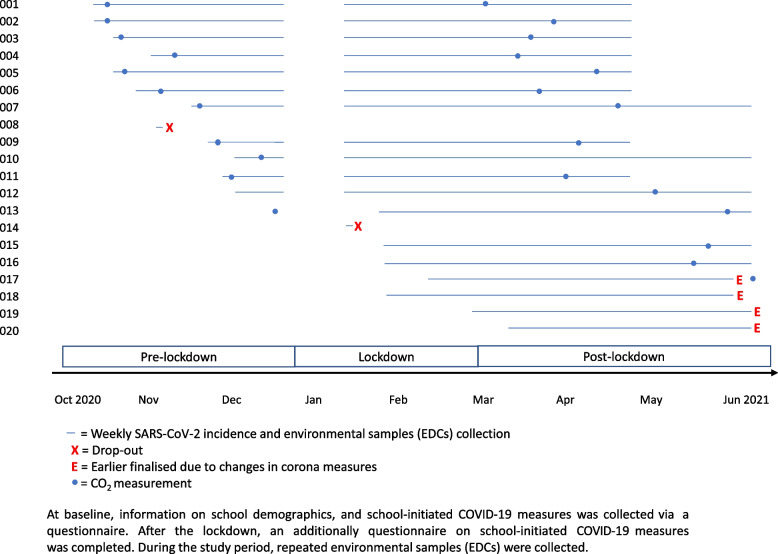
Study design and data-collection

For this prospective cohort study, schools were selected based on their size, educational provision and geographical location. Schools were categorized in theoretical schools (senior general secondary education and pre-university education) and vocational schools (pre-vocational secondary education). This distinction was made because group size and contact patterns might differ substantially between the two types. Schools were enrolled between October 2020 and March 2021 and follow-up ended in June 2021. Follow-up is defined as the time period that schools were participating in data-collection for this study, i.e. from enrollment until stop of participation or end the study in June 2021. Each participating school collected data on weekly number of reported SARS-CoV-2 infections among students and staff and school-absenteeism. In addition, details on school demographics were collected at baseline and schools completed questionnaires on school-initiated COVID-19 measures (Supplement Box S1 and S2). The study further included measurement of classroom CO_2_ concentrations at two time-points during follow-up, and repeated monitoring of SARS-CoV-2 air contamination using electrostatic dust collectors (EDC) during the entire follow-up period (Fig. 1).

### National COVID-19 policy

A detailed overview of the national COVID-19 policy in place during the study period can be found in Supplement Table S1. Most relevant for this study is the school closure on 16 December 2020 as part of a national lockdown with gradual reopening in 2021. Before the lockdown physical distancing (> 1.5 m) in schools was mandated for staff-staff and staff-student interactions, but not required in or outside school among children below 18 years. As of December 2020, mask mandates were in place for students and staff outside, but not inside the classroom. The national COVID-19 testing policy included PCR testing at municipal test centers for all symptomatic individuals. In December 2020, the testing policy was expanded to asymptomatic close contacts (> 15 min within 1.5 m of confirmed case). During the entire study period, schools were recommended to improve ventilation (e.g. by keeping doors and windows open as much as possible).

Between January 2021 and March 2021, schools provided full online education except for students in their final exam year and for students living under conditions unacceptable for home-schooling. This comprised about 20% of all students who attended school physically. From March 2021 onward, schools reopened to all students, but at half occupancy with alternating face-to-face and online education. During and after the lockdown physical distancing was expanded to student–student interactions and from March 2021 onwards, PCR testing was also available for all non-close contacts of confirmed cases, irrespective of symptoms.

The study period was divided into three categories based on the national COVID-19 policy: ‘pre-lockdown’ when schools operated at full occupancy (19 October—15 December 2020), ‘lockdown’ when about 20% of students attended school (18 January – 28 February 2021), and ‘post-lockdown’ when schools operated at half occupancy (1 March – 4 June 2021).

### School-initiated COVID-19 measures

In addition to the national COVID-19 policy, schools were advised to implement additional measures to enhance hygiene and minimize student–student and student-staff contacts. A set of recommendations was disseminated to all schools including suggestions for cohorting, measures to reduce crowding and hand hygiene protocols. The type and stringency of such measures implemented locally was determined by the school and varied between schools. Schools completed a baseline questionnaire on the type and number of school-initiated COVID-19 measures they had implemented. The questionnaire was repeated in March 2021 to capture any changes in local policy post-lockdown. The school-initiated COVID-19 measures were categorized into: 1) cohorting interventions, 2) (hand) hygiene measures, 3) student displacement reductions, and 4) measures promoting physical distancing. Cohorting interventions include separate walking routes, entrances, exits, start/stop moments class hours. Physical distancing includes fixed seating, extra breaks, and physical distancing (> 1.5 m). Student displacement reductions include dedicated classrooms for course hours and breaks. (Hand) hygiene includes use of splash guards, and access to disinfectant hand gel. For each category, the interventions implemented were summarized into a score per school; the “*school-initiated COVID-19 measures score*”. More details on school-initiated COVID-19 measures and scoring can be found in Supplement Table S2.

### CO_2_ concentration and threshold exceedance

As an indicator for ventilation rate per person, continuous CO_2_ concentrations were collected at the schools during teaching hours for one day pre- and post-lockdown. Three classrooms were selected that represented the different ventilation regimes present in the school. During the study period, there was additional attention for optimal ventilation and windows and doors of classrooms were frequently open. The procedure of monitoring indoor and outdoor CO_2_ concentrations was determined in a previous study by Zhang et al. 2022 [5]. CO_2_ concentrations were measured using HOBO® CO_2_ loggers (type: MX1102A; range: 0 to 5000 ppm; accuracy: ± 50 ppm). CO_2_ concentrations were monitored in classrooms on the front and back wall with a time interval of 30 s during the entire school day [5]. Pre-lockdown, the outdoor CO_2_ concentration was monitored at the entrance, both in the morning and in the afternoon, for 15 min. Post-lockdown, the outdoor CO_2_ concentration was monitored both at the entrance and in the courtyard, for the whole school day. To identify measurement errors, CO_2_ data points were converted to Z-scores. Z-scores > 3 were considered measurement errors and excluded for further analyses. Measurements from the different locations in the classroom were averaged per time-point. According to the Dutch Fresh Schools guideline [6], ventilation rate per person can be evaluated using indoor CO_2_ concentration as the indicator: ventilation rate per person is categorized into three levels: 1) Acceptable, 2) Good, and 3) Excellent, which correspond to an indoor CO_2_ concentration of 1) less than 800 ppm, 2) less than 550 ppm, 3) less than 400 ppm, above the outdoor CO_2_ concentration, respectively. The percent-time above each CO_2_ threshold was calculated by dividing the time when the difference between indoor and outdoor CO_2_ concentration exceeded 400 ppm, 550 ppm, and 800 ppm respectively, by the total measurement time. Breaks and unoccupied time (number of students = 0) were excluded. These calculated percent-time above each CO_2_ threshold (Acceptable, Good and Excellent) per classroom during occupancy were averaged over classrooms within a school to obtain a summary measure of percent-time CO_2_ exceedance per school.

### SARS-CoV-2 air contamination

To investigate possible airborne circulation of SARS-CoV-2 in the schools, longitudinal air monitoring was conducted through repeated collection of airborne settling dust samples in the schools between October 2020 until June 2021. At each included secondary school, several classrooms, the teachers’ office and the canteen were selected for longitudinal air monitoring. Classrooms were selected to represent different ventilation regimes resulting in the selection of three to six classrooms per school.

Airborne settling dust was sampled using Electrostatic Dust Collectors (EDCs), which were placed in the selected rooms [7]. EDCs were placed in holders at approximately 30 cm underneath the ceiling attached in the middle of the space away from open windows, ventilation grids and heaters. EDCs were collected and renewed after approximately four weeks of exposure or earlier when major changes in COVID-19 policies were implemented. Four to five repeated samples were collected within each school (Fig. 1) during a period of five months. EDCs were packed in minigrip™ bags and sent to the laboratory by post. Concurrently, schools were asked to complete a questionnaire about the occupancy and the change of ventilation regimes in the rooms. At the laboratory, electrostatic cloths were transferred into minigrip™ bags and stored frozen at -80℃ until further processing under biosafety laboratory (BSL)-2 + conditions [8]. RNA was extracted from EDC cloths based on the procedure by Biesbroek et al. and Wyllie et al. [9, 10] (See Supplement Box S3 for more details). RNA extracts were subsequently analyzed for the presence of SARS-CoV-2 RNA by RT-qPCR, targeting the E gene of SARS-CoV-2, as described previously [11]. As control for collection performance, we additionally investigated total bacterial load through 16S region V3-V4 qPCR as described by Fierer et al. [12].

### SARS-CoV-2 incidence in schools

Schools kept daily logs of reported SARS-CoV-2 infections among students and staff including information on the date of the positive test, and period of absence. Testing was performed in municipal testing facilities using a nasopharyngeal swab and subsequent RT-qPCR. During the lockdown period, schools only kept daily logs of those students and staff that were allowed inside school buildings during this period. Between 15 December 2020 and 17 January 2021 no data was collected due to the Christmas holidays and a transition period to the new and stricter national COVID-19 policy measures. Overall weekly SARS-CoV-2 incidence rate, and (median) weekly incidence rate per school were calculated by dividing the number of reported infections by the total number of students and staff members.

### Statistical analysis

The associations between each of the determinants (i.e. national COVID-19 policy, school-initiated COVID-19 measures, percent-time CO_2_ concentration exceedance, and SARS-CoV-2 air contamination) and the outcome SARS-CoV-2 incidence per school were investigated using a hierarchical approach;

First, the association between national COVID-19 policy and SARS-CoV-2 incidence was investigated. As potential confounders background municipal SARS-CoV-2 population incidence and school type (theoretical vs vocational) were included [13]. Second, per category, the school-initiated COVID-19 measure score was added as a continuous variable in the model. Third, we explored the association with CO_2_ concentration by adding the percent-time CO_2_ exceedance above threshold to the model in top-down order starting with the highest acceptable threshold (percent-time exceeding 800 ppm above the outdoor CO_2_ concentration). Percent-time CO_2_ exceedance was included as a continuous variable and as a dichotomized variable (< 10% versus ≥ 10% percent-time above threshold). Because of the strong correlation between CO_2_ concentration and class-occupancy (i.e. pre- vs post-lockdown), the model was stratified for lockdown period. In none of the environmental samples SARS-CoV-2 RNA was detected, and therefore this was not included in the model.

For all models we applied a Generalized Estimation Equations (GEE) analysis with negative binomial distribution and log link function. To take into account the correlation between repeated observations of SARS-CoV-2 incidence within schools, an autoregressive correlation matrix (AR1) was included. We report both crude and adjusted incidence rate ratios (IRR) and 95% confidence interval (95% CI) for each (group of) determinants. A *p*-value ≤ 0.05 was considered statistically significant. Statistical analyses were performed using SPSS version 26.0.0.1 and R version 4.0.3 [14, 15].

## Results

Between October 2020 and March 2021, 20 schools were enrolled in the study, of which two schools were lost-to-follow-up within two weeks after enrolment, and excluded from further analyses. Twelve schools were enrolled pre-lockdown, four schools during lockdown, and two schools post-lockdown. Total follow-up time of the 18 schools was 298 weeks, with median follow-up time per school of 18 weeks (range 10–22). The majority of the schools were located in urban settings (44.4%) and were theoretical schools (55.6%). School size ranged from 100–2015 students (Table 1).

**Table 1:** Characteristics of participating schools, follow-up period, and implemented school-initiated COVID-19 measures

**Participating schools (*n* = 18)**	n (weeks of follow-up)
Pre-lockdown	11 (60)
Lockdown	16 (63)
Post-lockdown	18 (175)
**School size**	median (range)
Students	806 (100–2015)
Staff	90 (26–208)
**School type**^**a**^	n (%)
Theoretical	10 (56)
Vocational	8 (44)
**Location**	n (%)
Urban	8 (44)
Suburban	6 (33)
Rural	4 (22)
**School-initiated COVID-19 measures**^**b**^	median score (range)
Cohorting interventions	2 (0–5)
(Hand) hygiene	6 (3–8)
Student displacement reductions	0 (0–2)
Physical distancing	5 (2–12)
**CO**_**2**_** exceedance**	Mean percentage of time (min–max)^c^
*Excellent (400 ppm)*	
Pre-lockdown	54 (1–86)
Post-lockdown	17 (0–43)
*Good (550 ppm)*	
Pre-lockdown	36 (0–73)
Post-lockdown	6 (0–25)
*Acceptable (800 ppm)*	
Pre-lockdown	13 (0–45)
Post-lockdown	1 (0–6)

^a^Theoretical schools included senior general secondary education and pre-university education. Vocational schools included pre-vocational secondary education

^**b**^For an overview of the school-initiated COVID-19 measures and calculation of the corresponding score we refer to Supplement Table S2

^c^Percent of time CO_2_ exceedance was calculated by dividing the time the CO_2_ concentration exceeded 400 ppm, 550 ppm, and 800 ppm above outdoor levels, respectively, by the total measurement time per occupied classroom. Mean estimates per school were averaged to obtain a summary estimate for all participating schools

### National COVID-19 policy

**Fig. 2:**
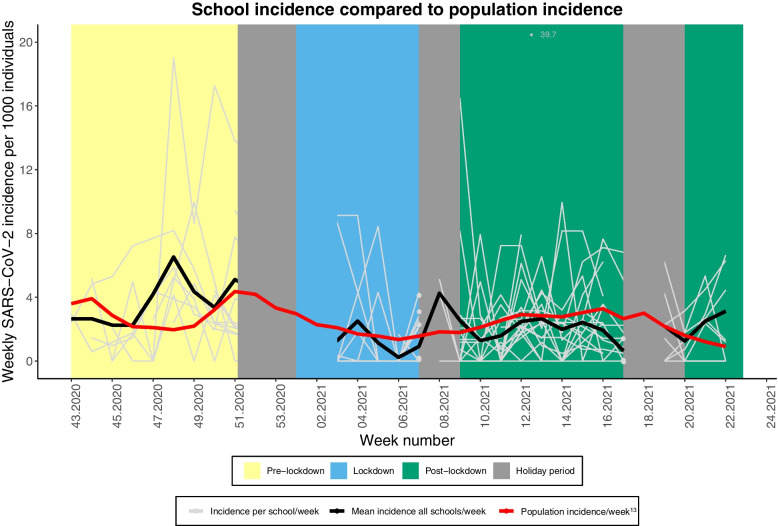
Dynamics of weekly SARS-CoV-2 incidence in schools and the Dutch general population between October 2020 and June 2021

The pattern over time of weekly SARS-CoV-2 incidence in schools was comparable to the general population incidence, although pre-lockdown and in week 7–9 of 2021 the mean school incidence exceeded the population incidence (Fig. 2). Weekly SARS-CoV-2 incidence rates per school were lower during and post-lockdown compared to pre-lockdown. SARS-CoV-2 school incidence significantly decreased during lockdown (IRR: 0.41; 95% CI: 0.21–0.80) and post-lockdown (IRR: 0.60; 95% CI: 0.39–0.93) compared to pre-lockdown, results were adjusted for school type and background municipal SARS-CoV-2 population incidence (Table 2).

**Table 2:** Crude and adjusted IRRs of the association between lockdown period and SARS-CoV-2 incidence within schools

	**Crude IRR (95%CI)**	**Adjusted**^**a**^** IRR (95%CI)**
**Lockdown period**^b^		
Pre-lockdown	1.00	1.00
Lockdown	0.34 (0.19–0.61)	0.41 (0.21–0.80)
Post-lockdown	0.57 (0.36–0.88)	0.60 (0.39–0.93)

*Abbreviations*: *IRR* Incidence rate ratio, *CI* Confidence interval

^a^Model was adjusted for school type (Theoretical reference, Vocational IRR 1.15 (0.86–1.55)), and background municipal SARS-CoV-2 population incidence (IRR 1.16 (0.92–1.45)

^b^ ‘Pre-lockdown’ period full occupancy, ‘lockdown ‘ period ≈20% occupancy, ‘post-lockdown’ period ≈ 50% occupancy. For a complete overview of the national COVID-19 policy within the different periods we refer to Supplement Table S1

### School-initiated COVID-19 measures

**Fig. 3:**
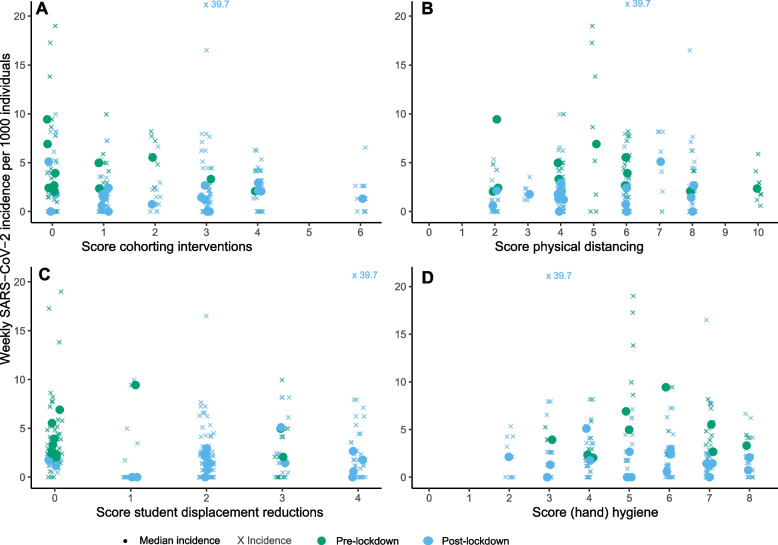
Weekly SARS-CoV-2 incidence per school for school-initiated COVID-19 measures.^*^: **A** cohorting interventions **B** physical distancing **C** student displacement reductions **D** (hand) hygiene. * Cohorting interventions include separate walking routes, entrances, exits, start/stop moments class hours. Physical distancing includes fixed seating, extra breaks, and physical distancing (> 1.5 m). Student displacement reductions include dedicated classrooms for course hours and breaks. (Hand) hygiene includes use of splash guards, and access to disinfectant hand gel. For an overview of all school-initiated COVID-19 measures and calculation of the total score we refer to Supplement Table S1

Figure 3 displays the different COVID-19 measure scores per school by weekly SARS-COV-2 incidence. In crude and adjusted analyses, there were no significant associations between any of the school-initiated COVID-19 measures (cohorting interventions; (hand) hygiene measures; student displacement reductions; measures promoting physical distancing) and SARS-CoV-2 school incidence (Table 3).

**Table 3:** Crude and adjusted IRRs of the association between school-initiated COVID-19 measures and SARS-CoV-2 incidence within schools

	**Crude IRR (95%CI)**	**Adjusted**^**a**^** IRR (95%CI)**
**School-initiated COVID-19 measures**^b^		
Cohorting interventions	0.93 (0.86–1.02)	1.04 (0.95–1.13)
(Hand) hygiene	0.97 (0.90–1.05)	0.95 (0.88–1.03)
Student displacement reductions	0.90 (0.76–1.06)	0.97 (0.85–1.12)
Physical distancing	1.06 (1.01–1.12)	1.07 (0.98–1.16)

*Abbreviations*: *IRR* Incidence rate ratio, *CI* Confidence interval

^a^Model was adjusted for lockdown period^c^, school type, and background municipal SARS-CoV-2 population incidence

^b^For an overview of the school-initiated COVID-19 measures and calculation of the corresponding score we refer to Supplement Table S2

^c^‘Pre-lockdown’ period full occupancy, ‘lockdown ‘ period ≈ 20% occupancy, ‘post-lockdown’ period ≈ 50% occupancy. For a complete overview of the national COVID-19 policy within the different periods we refer to Supplement Table S1

### CO_2_ concentration and threshold exceedance

**Fig. 4:**
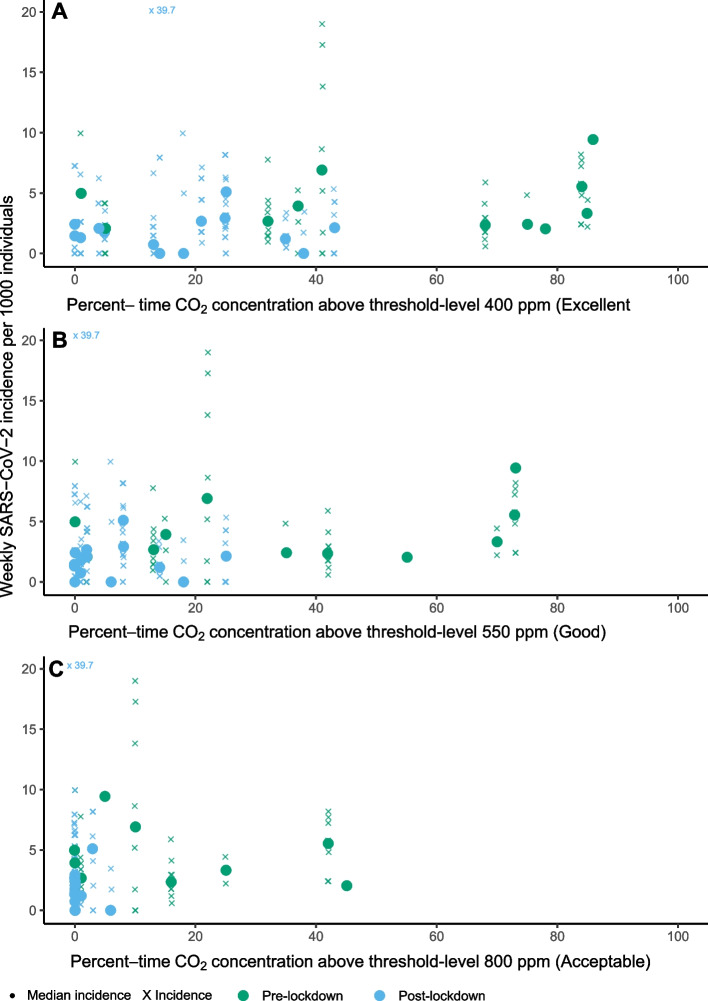
Weekly SARS-CoV-2 incidence per school by percent-time the difference between indoor and outdoor CO_2_ concentration above threshold-level 400 ppm (Excellent), 550 ppm (Good), and 800 ppm (Acceptable). Percent-time of CO_2_ concentrations above the threshold-level and weekly SARS-CoV-2 incidence per school. Crosses reflect individual weekly SARS-COV-2 incidence rates per school and dots reflect median values per school per period (pre-lockdown; post-lockdown). Percent-time of CO_2_ concentrations above threshold was calculated by dividing the time the difference between indoor and outdoor CO_2_ concentration exceeded 400 ppm, 550 ppm, and 800 ppm, respectively, by the total measurement time per occupied classroom. Measurements were averaged over classrooms to obtain a summary estimate per school

Thirteen schools (44 classrooms) were visited between October and December 2020 (pre-lockdown) to measure CO_2_ concentrations. Of these, 10 schools (37 classrooms) were visited a second time between March and June 2021 (post-lockdown). No schools were visited during the lockdown period. Additionally, three other schools (10 classrooms) were only visited post-lockdown. Figure 4 displays the percent-time of CO_2_ exceedance for each threshold and weekly SARS-CoV2 incidence per school. Across the three different thresholds (Acceptable; Good; Excellent), the percent-time exceedance was higher pre-lockdown, compared to post-lockdown (Fig. 4), which was strongly correlated with the occupancy levels in classrooms [16]. Starting with the highest threshold (Acceptable; 800 ppm), pre-lockdown, no statistically significant association between SARS-CoV-2 school incidence and percent-time CO_2_ exceedance above 800 ppm was observed on a continuous scale (IRR per 10% increase: 1.08; 95% CI: 0.95–1.22), nor when dichotomized (IRR for ≥ 10% versus < 10%: 1.46; 95% CI: 0.76–2.81) (Table 4). Post-lockdown, the difference between indoor and outdoor CO_2_ concentration almost never exceeded the 800 ppm threshold. For percent-time CO_2_ exceedance levels Good or Excellent, pre-lockdown the IRR point estimates were 1.10 (1.02–1.19) and 1.08 (95% CI: 1.00–1.16), respectively, whereas post-lockdown, the association could no longer be demonstrated (Table 4).

**Table 4:** Crude and adjusted IRRs of the association between percent-time the difference between indoor and outdoor CO_2_ concentrations exceeded the threshold of 800 ppm (Acceptable) and SARS-CoV-2 incidence within schools

	**Crude IRR (95%CI)**	**Adjusted**^**a**^** IRR (95%CI)**
**Pre-lockdown**^**c**^ Percent-time CO_2_ concentration exceeds threshold		
400 ppm	1.04 (0.97–1.12)^b^	1.08 (1.00–1.16)^b^
550 ppm	1.05 (0.97–1.14)^b^	1.10 (1.02–1.19)^b^
800 ppm	1.04 (0.96–1.14)^b^	1.08 (0.95–1.22)^b^
800 ppm < 10% time	reference	reference
800 ppm ≥ 10% time	1.42 (0.82–2.47)	1.46 (0.76–2.81)
**Post-lockdown**^**c**^ Percent-time CO_2_ concentration exceeds threshold		
400 ppm	1.01 (0.88–1.16)^b^	1.02 (0.87–1.20)^b^
550 ppm	0.88 (0.69–1.12)^b^	0.89 (0.68–1.15)^b^

*Abbreviations*: *IRR* Incidence rate ratio, *CI* Confidence interval

^a^Models were adjusted for school type, and background municipal SARS-CoV-2 population incidence

^b^Per 10% increase of time CO_2_ concentration exceeded threshold

^c^ ‘Pre-lockdown’ period full occupancy, ‘lockdown ‘ period ≈20% occupancy, ‘post-lockdown’ period ≈ 50% occupancy. For a complete overview of the national COVID-19 policy within the different periods we refer to Supplement Table S1. Only the IRR pre-lockdown is reported, because the difference between indoor and outdoor CO_2_ concentration almost never exceeded the 800 ppm threshold in the post-lockdown period

### SARS-CoV-2 air contamination

In total, 469 settling dust samples were collected in 18 schools. All samples were negative for SARS-CoV-2 E gene detection by RT-qPCR. All field blank samples tested also negative in RT-qPCR. Results of 16S qPCR showed that levels of total bacteria in school classrooms pre-lockdown were significantly higher compared to post-lockdown (mean Ct-value pre-lockdown 21.1 versus 22.3 post-lockdown, *p* < 0.001). For a complete overview of sample locations see Supplement Table S3.

## Discussion

This prospective cohort study aimed to gain insight in the factors influencing SARS-CoV-2 incidence among students and staff in secondary schools. We found a 40% reduction in weekly SARS-CoV-2 incidence rate in schools post-lockdown compared to pre-lockdown, suggesting a combined effect of the national COVID-19 policy measures introduced in the (post) lockdown period including reduced class-occupancy, stricter quarantine rules, and expanded access to SARS-CoV-2 testing. In this study, we found no additional effect of school-initiated COVID-19 measures and a consistent effect of percent-time CO_2_ exceedance could not be demonstrated. No SARS-CoV-2 RNA was detected during repeated air monitoring in the schools throughout the period of the study.

### National policy and School-initiated COVID-19 measures

Our findings confirm that during a period with low population immunity, the national policy of reduced class-occupancy combined with early identification of infected subjects through extensive testing and quarantine or isolation of (potentially) infected subjects is effective in lowering incidence in school settings. Secondary schools were also encouraged to implement self-initiated COVID-19 measures in addition to the national COVID-19 policy measures. Because large variation existed between schools in how these recommendations were implemented, we were able to investigate the effect of these school-initiated COVID-19 measures grouped by their primary aim of cohorting, (hand) hygiene, student displacement reductions, or physical distancing. None of these groups of interventions had a significant effect on reducing SARS-CoV-2 incidence in schools. Possible explanations are that most interventions were implemented only in designated areas of the school, and that many of these interventions require behavioral commitment from students and staff, which may be difficult to achieve, or the interventions are difficult to execute in crowded environments like schools. In line with this, two studies set in the United States showed no effect of school-initiated measures to promote stricter physical distancing in schools (from ≥ 3 feet to ≥ 6 feet) on SARS-CoV-2 incidence [17, 18]. A systematic review into COVID-19 measures in the school setting, concluded that a broad range of both national and school-initiated COVID-19 measures (e.g. lower occupancy, mask wearing, handwashing, ventilation) can have a positive impact on reducing SARS-CoV-2 transmission, but most (33 out of 38) studies were simulation studies, the effect of individual measures could not be disentangled, and overall the certainty of the evidence was low [4]. Our study adds empirical data on real-world effectiveness and ineffectiveness of (combined) mitigation measures in schools.

#### CO_2_ concentration and threshold exceedance

During the pandemic, schools were encouraged to improve ventilation using mechanical systems if present, or else by opening doors and windows. This advice was based on the notion that ventilation reduces aerosol density and therefore possibly transmission via the aerosol route. We hypothesized that more time with higher CO_2_ concentrations in classrooms, as a proxy for lower ventilation rate per person, would be associated with an increase in SARS-CoV-2 school incidence. Indeed, a further evaluation of the ventilation rates per classroom confirmed that the mean ventilation rates per classroom did not change before and after the lockdown, mostly likely resulting from the opening of doors and windows more frequently during both measurements, thereby creating sufficient ventilation [19, 20]. However, the mean ventilation rate *per person* increased as the average occupancy decreased [16]. Consequently, we observed a decrease in percent-time of CO_2_ exceedance post lockdown reflecting the net result of reduced class-occupancy (i.e. fewer sources of CO_2_ production) despite stable classroom ventilation rates. Yet, the association of CO_2_ exceedance with SARS-CoV-2 school incidence in our study was inconsistent: we found IRR point estimates that were similar or even in opposite direction for the different CO_2_ thresholds at 400, 550 and 800 ppm above outdoor CO_2_ levels before and after the lockdown. Our data suggest that effects of CO2 exceedance on SARS-CoV-2 incidence may depend on other classroom conditions besides occupancy levels that were different between pre-lockdown and post-lockdown period, and that may determine the dose of SARS-CoV-2 present in inhaled air (for instance difference in physical distance between subjects in class, difference in frequency of infectious subjects being present depending on testing policy). Our findings are in line with those of a modeling study that suggested ventilation has little effect on overall transmission risk [21]. Their study concluded that ventilation at room level can only have an effect on long range transmission and this requires presence of an infected person in the room with a very high emission rate of virus [21]. To our knowledge, no other field-studies are available that assessed the association between percent-time CO_2_ exceedance and SARS-CoV-2 incidence in secondary schools. One study reported a lower incidence in elementary schools after improvements in ventilation regime when compared to no improvements (RR: 0.61 95%CI:0.43–0.87), but the study did not include actual CO_2_ measurements, had a low response rate (11.6%) with missing information about ventilation improvements, and did not adjust for policy changes during the follow-up period [22].

#### SARS-CoV-2 air contamination

**Fig. 5:**
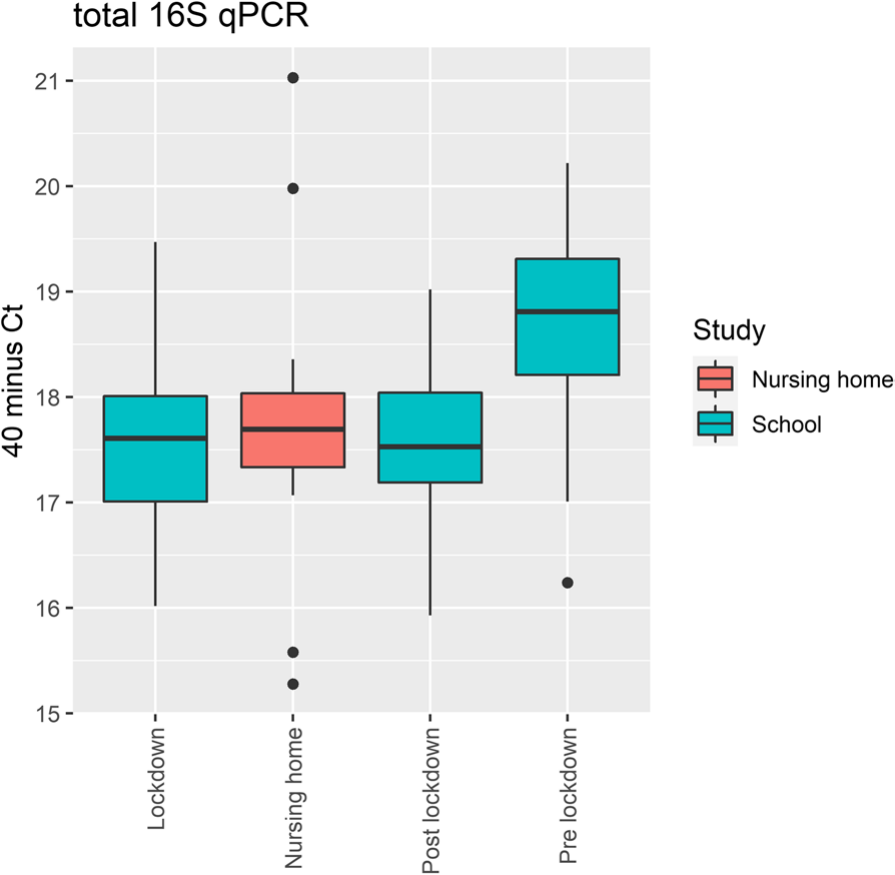
Total bacterial load 16S qPCR response for EDC samples collected in school classrooms during pre-lockdown, lockdown and post-lockdown period, and those for EDC samples collected in rooms of nursing home patients in isolation. Total bacterial loads are depicted as 40 minus Ct-value thus higher values express higher levels. The study underlying the nursing home data from this figure is reported in Linde et al. 2022 [23]

The absence of SARS-CoV-2 RNA in repeated air samples collected in school classrooms, canteen areas and teacher offices further suggest that major widespread airborne environmental contamination was uncommon in schools under the prevailing conditions at the time of the study with increased attention for ventilation, intensified screening and quarantine policies and reduced occupancy levels. This contradicts with findings in our previous studies in nursing homes, where SARS-CoV-2 RNA was detected in 83% of settling dust samples collected in rooms occupied by symptomatic SARS-CoV-2 patients and in 2.7% of samples collected from shared spaces such as the common living room [23]. Although we cannot confirm that infected subjects were present in the sampled classrooms during the study period, findings from our outbreak investigations in several of the participating schools during the pre-lockdown period confirmed presence of infected subjects at schools [24]. In contrast to nursing home patients, these subjects had minor or no symptoms which has been shown to be associated with lower viral loads, and likely also lower viral shedding as a consequence of that [25, 26]. Furthermore, presence of infected subjects in a classroom or other school areas was at most several hours per day. To rule out that differences in SARS-CoV-2 air contamination between nursing homes and schools could be explained by differences in EDC collection capacity, we compared the total bacterial load measured by 16S qPCR in school classrooms to those of the nursing home rooms and found that levels during and post-lockdown were similar to those in the nursing home rooms (Fig. 5). Moreover, total bacteria levels from pre-lockdown school samples were significantly higher compared to post-lockdown, supporting that EDCs attached at the classroom ceiling are able to capture and correlate with the number of sources present at lower height under the prevailing conditions. This corroborates findings of previous studies applying EDCs in elementary schools in a similar set-up showing associations between levels of endotoxin (measure for gram-negative bacteria) and higher occupancy [27], and between levels of cat and dog allergens and the percentage of children in the classroom having a cat or dog at home [28]. Combined, these data support the validity of EDC sampling to measure SARS-CoV-2 air contamination. We conclude that levels of air contamination in schools were too low to detect in our study.

#### Strengths and limitations

The major strength of this study is our prospective study design and extensive data collection on weekly SARS-CoV-2 incidence, implemented school-initiated COVID-19 measures, CO_2_ concentrations and SARS-CoV-2 air contamination. This allowed us to investigate direct associations between SARS-CoV-2 incidence and various preventive COVID-19 measures, as well as percent-time CO_2_ exceedance and air contamination in schools. However, some limitations should be addressed: first, reporting on SARS-CoV-2 infections to schools may have been incomplete and resulted in underestimation of the actual incidence. Second, detailed information about the location of actual transmission (in or outside school environment) could not be obtained. Results from our outbreak investigations conducted in some of the participating schools suggest that clusters of infections resulted from both within school transmission and from multiple introductions [24]. Third, the sample size in this study was not sufficient to determine statistical significance of small effect sizes. Fourth, the CO_2_ concentration was only measured for one or two days during the study period in a selection of classrooms per school and thus might not be representative for the entire study period and school. We selected the classrooms and days to represent the different ventilation regimes covering within school variation, but in most of the classrooms the operation of mechanical ventilation (if available) was affected by opening windows and doors at the time of the measurements. Therefore, the effect of different ventilation regimes on SARS-CoV-2 incidence could not be evaluated and our results apply to school settings where windows and doors of classrooms are frequently open. Probably, if doors and windows were closed more often, this would have resulted in CO_2_ thresholds being exceeded more frequently.

## Conclusions

In conclusion, during a period with low population immunity to SARS-CoV-2 and increased attention to ventilation, a national COVID-19 policy including reduced class-occupancy, expanded quarantine and testing of contacts reduced the incidence of SARS-CoV-2 in Dutch secondary schools. Additional school-initiated COVID-19 measures did not reduce SARS-CoV-2 incidence. Under conditions where CO_2_ levels remained below acceptable thresholds for most of the time, a consistent effect of percent-time CO_2_ exceedance on SARS-CoV-2 incidence could not be confirmed and effects may depend on other classroom conditions (i.e. difference in occupancy, difference in physical distance between subjects in class, difference in frequency of infectious subjects being present depending on testing policy). Widespread SARS-CoV-2 air contamination could not be demonstrated in schools under the prevailing conditions during the study.

## Supplementary Information

**Additional file 1:  Box S1.** Survey baseline characteristics school. **Box S2.** Survey school-initiated COVID-19 measures. **Table S1.** National COVID-19 policy during the study period (October 2020 – June 2021) for each lockdown period. **Table S2.** School-initiated COVID-19 measures and corresponding scores. **Box S3.** Laboratory analysis of settling dust samples. **Table S3.** Results of SARS-CoV-2 RT-PCR in settling dust samples in secondary schools.

## Data Availability

The datasets used and analyzed during the current study are available from the corresponding author on reasonable request.

## Abbreviations

EDC: Electrostatic Dust Collectors
IRR: Incidence Rate Ratios
GEE: Generalized Estimating Equation
CI: Confidence Interval
