# Supplementary material for: SARS-CoV-2 incidence in secondary schools; the role of national and school-initiated COVID-19 measures
Source: BMC Public Health. 2023 Jun 27;23:1243. doi: 10.1186/s12889-023-16146-0 (PMC10294357; doi:10.1186/s12889-023-16146-0)
Supplement: Supplementary file 1 — Additional file 1: Box S1. Survey baseline characteristics school. Box S2. Survey school-initiated COVID-19 measures. Table S1. National COVID-19 policy during the study period (October 2020 – June 2021) for each lockdown period. Table S2. School-initiated COVID-19 measures and corresponding scores. Box S3. Laboratory analysis of settling dust samples. Table S3. Results of SARS-CoV-2 RT-PCR in settling dust samples in secondary schools. [file 12889_2023_16146_MOESM1_ESM.docx]

**Supplementary material**

**Table of contents**

1. Methods
   1. Box S1 Survey baseline characteristics school……………………………………………Page 2
   2. Box S2 Survey school-initiated COVID-19 measures……………………………………Page 3
   3. Table S1 National COVID-19 policy during the study period (October 2020 – June 2021)

for each lockdown period………………………………………………........................... Page 5

- 1. Table S2 School-initiated COVID-19 measures and corresponding scores……………... Page 6
  2. Box S3 Laboratory analysis of settling dust samples...………………………………….. Page 7

1. Results
   1. Table S3 SARS-CoV-2 PCR in settling dust samples in secondary schools……………. Page 8
   2. **Box S1 Survey baseline characteristics school**

**School ID**

|  |  |
| --- | --- |

1. Number of students

|  |  |  |  |
| --- | --- | --- | --- |

1. Number of teaching staff

|  |  |  |
| --- | --- | --- |

1. Number of other staff, contact with students*

|  |  |  |
| --- | --- | --- |

1. Number of other staff, no contact with students*

|  |  |  |
| --- | --- | --- |

* Contact with students is defined as having contact with (one or more) students several times a day as part of the regular activities.

1. Number of seperate school buildings over which the students are divided

|  |  |
| --- | --- |

1. Type of school

- Public
- Specific education
- Special education

1. Education offered (multiple answers possible)

- Learning support education (lwoo)
- Practical labour-oriented education (Praktijkonderwijs)
- Pre-vocational secondary education (VMBO)
- Senior general secondary education (HAVO)
- Pre-university education (VWO)
- Gymnasium/Technasium

1. Type of specific education;

- montessori
- jenaplan
- freinet
- dalton

1. Maxiumum number of student per class/subject that is educated in one classroom (except physical education)

|  |  |
| --- | --- |

1. Is there a fixed seating plan for the students per subject?

- Yes
- No

**1.2 Box S2 Survey school-initiated COVID-19 measures**

1. Does your school adhere to the COVID-19 protocol of the VO-council?

- Yes, all proposed measures
- Yes, part of the proposed measures
- No

1. Did your school implement one way walking routes?

- Yes, all corridors/common areasJa, in alle gangen/gedeelde ruimtes
- Yes, in a selection of corridors/common areas
- No

1. Are there seperate entrances and exits?

- Yes
- No

1. What method does your school use with regard to classroom changes?

- The students change classrooms in between lessons, teachers stay (as much as possible) in the same classroom
- Teachers change classrooms in between lessons, students stay (as much as possible) in the same classroom

1. Which measures did your school implement to reduce crowding? (multiple answers possible)

- Different start and stopping times for course hours
- Spreading timeslots for breaks
- Extra break rooms outdoor
- Extra break rooms indoor
- (selection of) students stay in classroom during breaks

1. Is it possible to keep 1.5 meter physical distance in the teachers lounge?

- Yes
- No
- Partly

1. Is physical distancing (1.5 meter) encouraged in students?

- Yes
- No

1. What is the seating plan for students in classrooms?

- Tables together in rows of two or three
- Seperate seating (space between tables)
- Other, namely...

1. Is it requested that students wear a mouth-nose mask?

- Yes, in corridors/common areas
- Yes, in corridors/common areas and during teaching hours
- No

1. Is it requrested that teachers wear a mouth-nose mask?

- Yes, in corridors/common areas
- Yes, in corridors/common areas and during teaching hours
- No

1. Does your school has a splash guard for teachers?

- Yes, in all classrooms
- Yes, in a selection of classrooms
- No

1. Where is the disinfectant hand gel available? (multiple answers possible)

- In classrooms
- At the school entrances
- In toilet roomsIn toiletruimtes
- In the cafetaria
- In the teachers lounge
- Other; ………………….

1. Are students asked to disinfect their hands upon entering the classroom?

- Yes
- No

1. Are teachers asked to wipe their own table with soap/cleaning agent upon entering the classroom?

- Yes
- No

**1.3 Table S1 National COVID-19 policy during the study period (October 2020 – June 2021) for each lockdown period**

| **Lockdown period** | **Policy** |
| --- | --- |
| Pre-lockdown  (19 October – 15 December 2020) | - National COVID-19 prevention measures included stay-home orders for individuals with respiratory complaints or fever - Physical distancing (>1.5 meter) for people aged 18 years or older - Permanently opening the windows and doors in absence of mechanical ventilation - Funds to purchase new mechanical ventilation systems - Until 30 November 2020 PCR tests available at municipal testing facilities for symptomatic individuals - From 1 December 2020 onwards PCR tests available at municipal testing facilities for asymptomatic close (school) contacts. - From 1 December onwards mask mandates within the school building, except the classrooms - Full occupancy without 1.5m distancing between students |
| Lockdown  (18 January – 28 February 2021) | - National COVID-19 prevention measures included stay-home orders for individuals with respiratory complaints or fever - 1.5m distance between students or bubbles - Permanently opening the windows and doors in absence of mechanical ventilation - PCR tests available at municipal testing facilities for asymptomatic close (school) contacts - Mask mandates within the school building, except in the classrooms. - 1.5m distance between students - Only exam students and for students living under conditions unacceptable for home-schooling |
| Post-lockdown  (1 March – 11 June 2021) | - National COVID-19 prevention measures included stay-home orders for individuals with respiratory complaints or fever - Permanently opening the windows and doors in absence of mechanical ventilation. - PCR tests available at municipal testing facilities for all (school) contacts. - Mask mandates within the school building, except in the classrooms. - 1.5m distance between students or bubbles - Half occupancy (e.g. different school buildings, alternating groups present within the school) |

**1.4 Table S2 School-initiated COVID-19 measures and corresponding scores**

|  | 0 points | 1 points | 2 points |
| --- | --- | --- | --- |
| **Cohorting interventions** |  |  |  |
| One way walking routes | No | Yes, partly | Yes |
| Separated use of entrances and exits | No | Yes, partly | Yes |
| Cohorts have different start and stopping times for course hours | No | NA | Yes |
| **(Hand) hygiene** |  |  |  |
| Use of splash guards for teachers | No | Yes, partly | Yes |
| Policy for encouraging hand hygiene students when entering the classroom | No | NA | Yes |
| Policy for work spot hygiene teachers when entering the classroom | No | NA | Yes |
| Access to disinfectant hand gel | 3 or less locations* | 4 locations | 5 or 6 locations |
| **Student displacements reductions** |  |  |  |
| Students stay in one classrooms for multiple course hours | No | Partly | Yes |
| Students stay in classroom during breaks | No | NA | Yes |
| **Physical distancing** |  |  |  |
| Fixed seating map for students in classroom | No | NA | Yes |
| Separated seats | No | NA | Yes |
| Extra break rooms indoor | No | NA | Yes |
| Extra break rooms outdoor | No | NA | Yes |
| Spreading timeslots for breaks | No | NA | Yes |
| Physical distancing (>1.5m) students | No | NA | Yes |

**Number of shared locations in school where disinfectant hand gel was freely available.*

*Maximum score for Cohorting interventions was 6 points; (Hand) hygiene 8 points; Student displacement reductions 4 points; Physical distancing 12 points.*

**1.5 Box S3 Laboratory analysis of settling dust samples**

At the laboratory, cloths from the settling dust samplers were removed from the EDC and transferred into minigrip^TM^ bags and stored frozen at -80℃ until further processing under biosafety laboratory (BSL)-2+ conditions [1]. RNA was extracted from EDC cloths based on the procedure by Biesbroek et al. and Willy et al [2,3]. Samples were thawed and transferred to tube containing 10 ml lysis buffer (LGC Genomics, Berlin, Germany), vortexed on a multipurpose vortex mixer for 10 minutes and centrifuged for 15 minutes at 4000 rpm then 800 μl supernatant was transferred to tube containing 0.4 gram glass beads in lysis buffer (Sigma-Aldrich, St Louis, Mo), 1000 μl phenol (Sigma-Aldrich, St Louis, Mo) and 5 μl PDV virus as control. Samples were mechanically disrupted through two cycles of 1 minute of vortex (24 plate 6.5m/s), 5 minutes on ice and 1 minute of vortex, before centrifugation at 8200 rcf for 10 minutes. The aqueous DNA phase, 650 μl supernatant, was then transferred to Eppendorf tubes pre-filled with 10 μl magnetic bead suspension (LGC Genomics, Berlin, Germany) and 1300 μl binding buffer (LGC Genomics, Berlin, Germany). Following 30 minutes of incubation at RT in mixing machine 800 rpm/min, the beads were washed with 200 μl wash buffers 1 and 2 (LGC Genomics, Berlin, Germany), and then magnetic beads were dried at 55℃ for 20 minutes. Subsequently samples were resuspended in 50 μl elution buffer (LGC Genomics, Berlin, Germany) and incubated for 15 minutes at 55℃. Supernatant was transferred and aliquoted into two tubes and stored frozen at -80℃ till transport to Erasmus MC. Presence of SARS-CoV-2 RNA was tested through a SARS-CoV-2 RNA RT-PCR, targeting the E gene of SARS-CoV-2, as described previously [4].

**2.1 Table S3 Results of SARS-CoV-2 RT-PCR in settling dust samples in secondary schools**

|  | | | | |  |
| --- | --- | --- | --- | --- | --- |
|  | **EDC - Settling dust** | | | | |
| **School ID** | **Period 1**  **n pos/N^#^** | **Period 2**  **n pos/N** | **Period 3**  **n pos/N** | **Period 4**  **n pos/N** | **Period 5**  **n pos/N** |
| 1 | 0/8 | 0/8 | 0/8 | 0/8 | 0/8 |
| 2 | 0/8 | 0/8 | 0/8 | 0/8 | 0/8 |
| 3 | 0/7 | 0/7 | 0/7 | 0/6 | 0/7 |
| 4 | 0/7 | 0/7 | 0/7 | 0/7 | NO |
| 5 | 0/6 | 0/6 | 0/6 | 0/6 | 0/6 |
| 6 | 0/7 | 0/7 | 0/7 | 0/7 | 0/7 |
| 7 | 0/6 | 0/6 | 0/6 | 0/6 | NO |
| 9 | 0/5 | 0/5 | 0/5 | 0/5 | NO |
| 10 | 0/6 | 0/6 | 0/6 | 0/6 | NO |
| 11 | 0/6 | 0/6 | 0/6 | 0/6 | NO |
| 12 | 0/8 | 0/8 | 0/8 | 0/8 | NO |
| 13 | 0/5 | 0/5 | 0/5 | 0/5 | 0/5 |
| 15 | 0/8 | 0/8 | 0/8 | 0/7 | NO |
| 16 | 0/4 | 0/4 | 0/4 | 0/4 | NO |
| 17 | 0/5 | 0/5 | 0/5 | 0/5 | NO |
| 18 | 0/6 | 0/6 | 0/6 | 0/6 | NO |
| 19 | 0/8 | 0/8 | 0/8 | NO | NO |
| 20 | 0/6 | 0/6 | 0/6 | NO | NO |

NO = samples were not obtained

# number of samples testing positive for SARS-CoV-2 RNA / total number of samples (n pos/N)
